# HisCoM-mimi: software for hierarchical structural component analysis for miRNA-mRNA integration model for binary phenotypes

**DOI:** 10.5808/GI.2019.17.1.e10

**Published:** 2019-03-31

**Authors:** Yongkang Kim, Taesung Park

**Affiliations:** 1 Department of Statistics, Seoul National University, Seoul 08826, Korea; 2 Interdisciplinary Program in Bioinformatics, Seoul National University, Seoul 08826, Korea

**Keywords:** integration analysis, miRNA, miRNA database, mRNA

## Abstract

To identify miRNA-mRNA interaction pairs associated with binary phenotypes, we propose a hierarchical structural component model for miRNA-mRNA integration (HisCoM-mimi). Information on known mRNA targets provided by TargetScan is used to perform HisCoM-mimi. However, multiple databases can be used to find miRNA-mRNA signatures with known biological information through different algorithms. To take these additional databases into account, we present our advanced application software for HisCoM-mimi for binary phenotypes. The proposed HisCoM-mimi supports both TargetScan and miRTarBase, which provides manually-verified information initially gathered by text-mining the literature. By integrating information from miRTarBase into HisCoM-mimi, a broad range of target information derived from the research literature can be analyzed. Another improvement of the new HisCoM-mimi approach is the inclusion of updated algorithms to provide the lasso and elastic-net penalties for users who want to fit a model with a smaller number of selected miRNAs and mRNAs. We expect that our HisCoM-mimi software will make advanced methods accessible to researchers who want to identify miRNA-mRNA interaction pairs related with binary phenotypes.

**Availability:** HisCoM-mimi is available at http://statgen.snu.ac.kr/software/hiscom-mimi/.

## Introduction

miRNA is a well-known form of noncoding RNA that affects biological mechanisms by regulating the expression of target mRNA. Many researchers have found that cancer cells and normal cells exhibit different inhibition mechanisms, suggesting that miRNAs could be used as biological markers for the diagnosis of cancer [1-3]. In our previous study, we presented a hierarchical structural component model (HisCoM-mimi) to find the miRNA-mRNA interaction pairs associated with binary phenotypes that could be candidates for cancer diagnosis biomarkers with an interpretable biological inhibition mechanism. Recently, many findings regarding the target mRNAs of miRNAs have been incorporated into various databases. TargetScan is one such database with recently updated findings [4]. The basic principle used by TargetScan to predict the mRNAs that miRNAs target for inhibition is to compare the sequences of untranslated mRNA regions to those of miRNAs [5]. However, many studies have shown that miRNAs select their target mRNAs based not only on the similarity of sequences, but also on other structural findings [6]. Thus, miRNA-mRNA integration analysis requires experimental confirmation of which mRNAs are truly inactivated by miRNAs. MiRTarBase is a database that collects experimental findings [6]. In our previous study, we only used TargetScan to find pairs of miRNA-mRNA relationships [7]. To enable researchers to utilize more flexibly information regarding the target mRNAs inhibited by miRNAs, we added miRTarBase database information to our software.

## Implementation

**Fig. 1.**
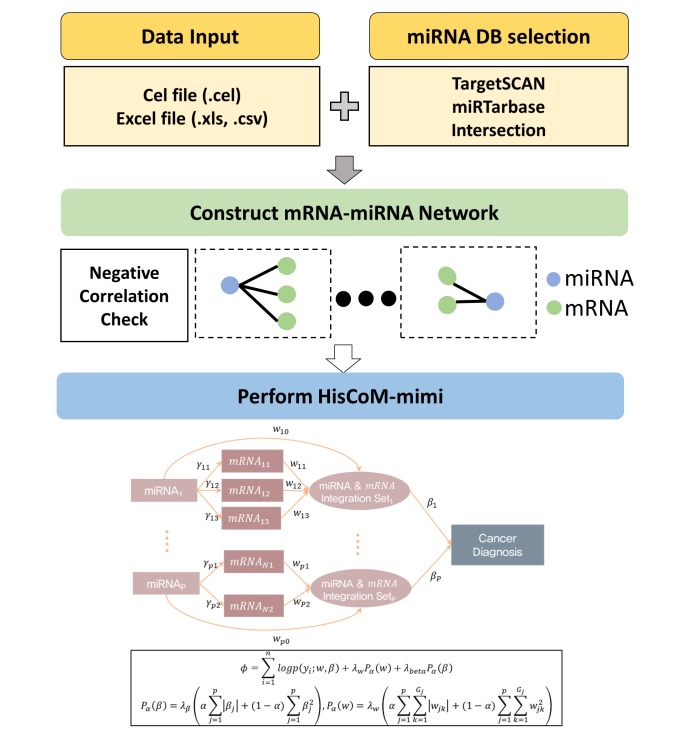
Workflow of HisCoM-mimi. The hierarchical structural component analysis workflow for the HisCoM-mimi application is shown. HisCoM-mimi first constructs an mRNA-miRNA network that connects negatively correlated miRNAs and target mRNAs. It then estimates the model parameters by maximizing the objective function via the alternating least square method with double-penalty functions for the coefficients. HisCoM-mimi, hierarchical structural component model for miRNA-mRNA integration.

Fig. 1 shows the hierarchical structural component analysis workflow for the HisCoM-mimi application, which requires miRNA and mRNA expression datasets and additional files (phenotype and covariates). The program now accepts two formats (miRNA and mRNA CEL files or an Excel-type expression dataset).

Next, miRNA-mRNA networks are constructed by combining the miRNA database information and correlation coefficients computed based on the user-entered datasets. Users can select an miRNA database in three ways: TargetScan results, miRTarBase results, and the intersection of both databases. The user can define the filtering network criteria by two options: (1) the choice of the databases and (2) a p-value threshold for the correlation coefficients between miRNAs and mRNAs.

After constructing an mRNA-miRNA integration set, HisCoM-mimi can be performed. HisCoM-mimi can accommodate not only the ridge penalty, but also the lasso and elastic-net penalties, which result in reduced computing time and a smaller number of nonzero coefficients [8, 9]. A cross-validation procedure is necessary to find the optimal penalties that maximize the log likelihood of the validation set [7].

## Conclusion

In this paper, we introduced our HisCoM-mimi software for miRNA-mRNA integration analysis. The current HisCoM-mimi application can use both the TargetScan and miRTarBase databases. Furthermore, in addition to the ridge penalty, HisCoM-mimi can accommodate lasso and elastic-net penalties.
